# Supplementary material for: Identification of photoperiod-induced specific miRNAs in the adrenal glands of Sunite sheep (Ovis aries)
Source: Front Vet Sci. 2022 Jul 22;9:888207. doi: 10.3389/fvets.2022.888207 (PMC9354845; doi:10.3389/fvets.2022.888207)
Supplement: Supplementary file 1 [file Data_Sheet_1.ZIP › Supplementary file/miRDeep2 file.zip!/miRDeep2 file/Know_Structure/SPLP42A2/oar-mir-487b.pdf]

miRBase precursor : oar-mir-487b
Total read count : 4089
oar-miR-487b-5p read count : 130
oar-miR-487b-3p read count : 3950
remaining reads : 9

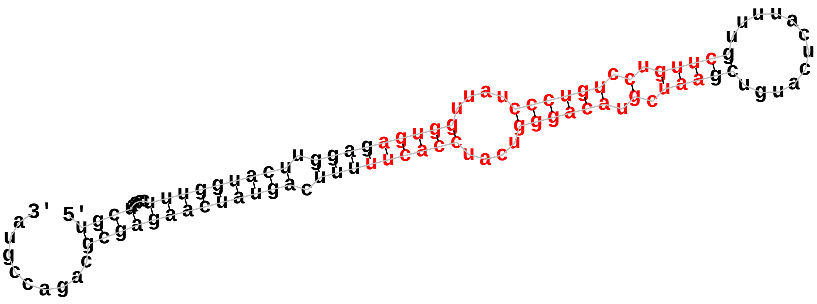

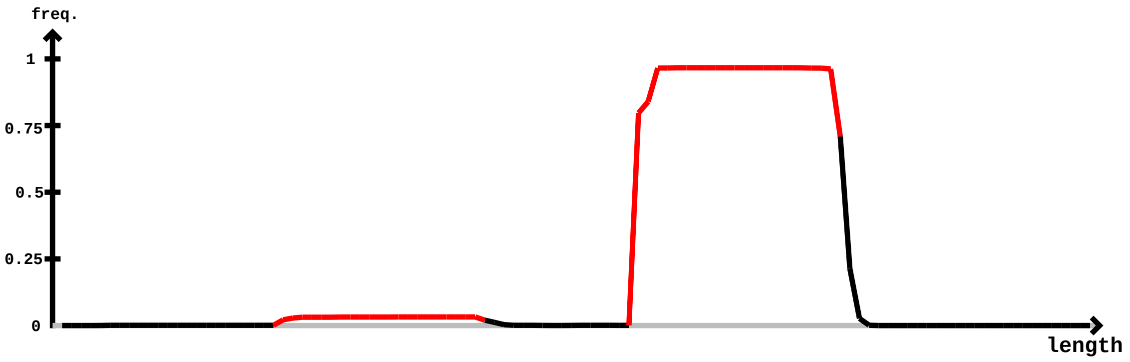

oar-miR-487b-3p

oar-miR-487b-5p

5'- ugcuaaccuuugguacuuggagagugguuaucccuguccuguucguuuuacucaugucgaaucguacagggucauccacuuuucaguaucaagagcgcagaccgua -3'	exp		
.(((....(((((((((.(((((((((....((((((.(.(((((............))))).).))))))....))))))))).))))))))))))..........	reads	mm	sample
...uaaccuuugguacuuggaga..	1	0	seq
....aaccuuugguacuuggaga..	3	0	seq
......................agugguuaucccuguccuguu..	47	0	seq
......................agugguuaucccuguccuguuc...	21	0	seq
......................agugguuaucccuguccuguucg..	11	0	seq
......................agugguuaucccuguccuguucgu...	5	0	seq
.......................gugguuaucccuguccuguu..	3	0	seq
.......................gugguuaucccuguccuguuc...	9	0	seq
.......................gugguuaucccuguccuguucg..	15	0	seq
.......................gugguuaucccuguccuguucgu...	4	0	seq
........................ugguuaucccuguccuguuc...	3	0	seq
........................ugguuaucccuguccuguucg..	9	0	seq
........................ugguuaucccuguccuguucgu...	1	0	seq
............................uaucccuguccuguucguuuu..	2	0	seq
............................uaucccuguccuguucguuuua...	1	0	seq
..................................uguccuguucguuuuacucaugu..	1	0	seq
....caugucgaaucguacagggucauccacu...........................	1	0	seq
....augucgaaucguacagggucauc...............................	1	0	seq
....augucgaaucguacagggucauccacuu..........................	1	0	seq
....aaucguacagggucaucc..............................	3	0	seq
....aaucguacagggucaucca.............................	1	0	seq
....aaucguacagggucauccac............................	11	0	seq
....aaucguacagggucauccacu...........................	1010	0	seq
....aaucguacagggucauccacuu..........................	1932	0	seq
....aaucguacagggucauccacuuu.........................	294	0	seq
....aaucguacagggucauccacuuuu........................	4	0	seq
....aucguacagggucaucc..............................	1	0	seq
....aucguacagggucauccac............................	1	0	seq
....aucguacagggucauccacu...........................	16	0	seq
....aucguacagggucauccacuu..........................	61	0	seq
....aucguacagggucauccacuuu.........................	85	0	seq
....aucguacagggucauccacuuuu........................	12	0	seq
....ucguacagggucauccacu...........................	7	0	seq
....ucguacagggucauccacuu..........................	31	0	seq

```
                                                          oar-miR-487b-3p
                              oar-miR-487b-5p
ugcuaaccuuugguacuuggagagugguuaucccuguccuguucguuuuacucaugucgaaucguacagggucauccacuuuuucaguaucaagagcgcagaccgua

.............................................................ucguacagggucauccacuuu.........................    384    0    seq
.............................................................ucguacagggucauccacuuuu........................    88     0    seq
.............................................................ucguacagggucauccacuuuuu.......................    5      0    seq
...............................................................guacagggucauccacu...........................    2      0    seq
...............................................................guacagggucauccacuu..........................    2      0    seq
```
